# Evaluation of a Novel Zonular Tension Restoring Accommodating Silicone IOL Design: Pilocarpine and Cyclopentolate-Induced Effect 20 Months after Implantation

**DOI:** 10.1155/2021/4797851

**Published:** 2021-11-29

**Authors:** Nino Hirnschall, Raoul Paolo D. Henson, Jay Marianito S. Vicencio, Andrew L Angeles, Oliver Findl, Andrew Phillips

**Affiliations:** ^1^VIROS-Vienna Institute for Research in Ocular Surgery, A Karl Landsteiner Institute, Hanusch Hospital, Vienna, Austria; ^2^Department of Ophthalmology and Optometry, Kepler University Hospital GmbH, Johannes Kepler University Linz, Linz, Austria; ^3^Clinica Henson Eye, Ear, Nose, and Throat Foundation, Angeles, Philippines; ^4^St Lukes Medical Center, Quezon, Philippines; ^5^Angeles University Foundation Medical Center, Angeles, Philippines; ^6^Phillips Eye Center, Los Angeles, CA, USA

## Abstract

**Purpose:**

To investigate a novel zonular-stress restoring accommodating 1-piece silicone IOL. *Setting*. Angeles City, Philippines.

**Design:**

Prospective randomized bilateral study.

**Methods:**

Each patient received a study IOL (ActaLens™, Emmetrope, La Canada, CA) in one eye and a control IOL (CrystaLens® AO, B&L, USA, or an AcrySof IQ®, Alcon, USA) in the contralateral eye to allow for intraindividual comparison. At the 20-month follow-up, two measurement days were set to measure all eyes before and after instilling 2% pilocarpine on the first day and 1% cyclopentolate on the second measurement day using an optical biometry device (Lenstar, Haag-Streit AG, Switzerland), respectively. PCO was graded by two examiners independently at the slit lamp.

**Results:**

In total, 16 eyes of 8 patients were included. In the study group and the control group, the pilocarpine-induced ACD shift was 0.32 mm (SD: 0.12) (*p*=0.014) and 0.04 mm (SD: 0.16) (*p*=0.854), respectively. In the study group and the control group, the mean cyclopentolate-induced ACD shift was 0.14 (SD: 0.06) (*p*=0.014) and 0.03 mm (SD: 0.03) (*p*=0.181), respectively. PCO and Nd : YAG rates were higher in the study group, but differences were not found to be significant (AcrySof vs. ActaLens *p*=0.100 and CrystaLens vs. ActaLens *p*=0.174).

**Conclusion:**

The investigated IOL is a novel concept for an accommodating IOL, and results showed a moderate pilocarpine-induced forward shift of the IOL 20 months following implantation. For all patients, the investigated IOL seems to have a higher PCO rate compared to standard monofocal IOLs.

## 1. Introduction

Within the last decades, patients' demand for spectacle independence after cataract surgery has increased rapidly. Modern intraocular lenses (IOLs) and modern IOL power calculation allow a significantly improved postoperative uncorrected distance visual acuity (and quality), but it is still not possible to restore accommodation after cataract surgery. Accommodation depends on biometrical changes, such as changes in lens thickness and anterior chamber depth, but mainly on the changes in the curvature of the lens [1]. Spectacle dependence can be reduced by either monovision or multifocal IOLs. Both attempts include an optical compromise; in the case of the former, there will be reduced stereopsis and possible discomfort due to the anisometropia. In the case of the latter, there will be mainly the loss in contrast vision and halos and glare, resulting in visual quality being imperfect at all distances. Another option is to use an IOL that accommodates. Most accommodating IOLs have one single optic and use the optic-shift principle. The original proposed mechanism for movement in these optic-shift IOLs was a change in vitreous pressure during accommodation which would vault the optic forward. It was shown that the back surface of the natural crystalline lens moves posteriorly during accommodation and that accommodation persists in young phakic patients following vitrectomy [2]. The alternatively proposed working principle is that the relaxation of the zonules induced by ciliary muscle contraction causes relaxation of the elastic capsular bag. As a consequence, circumferential compression is applied onto the haptics and a forward movement of the IOL is induced. Until today, none of the investigated trials on so-called accommodating IOLs showed relevant and persistent accommodation, and all studies were considered to have a severe performance bias [3, 4]. Accommodating IOLs have also been proposed that increase optical power by fluid shifts in the implanted optic, with the driving force, again proposed to be capsular elasticity.

The aim of this study was to investigate a novel concept of an accommodating IOL that introduces a novel mechanism of restoring zonular tension. With this design, the accommodating force acting on the IOL is intended to come from the elastomeric hinge of the IOL rather than capsular elasticity.

## 2. Materials and Methods

This case series with bilateral comparison included patients who were scheduled for bilateral cataract surgery. Inclusion criteria were patients between 50 and 75 years of age with best-corrected visual acuity (BCVA) of less than or equal to 20/50 and greater than or equal to 20/200. Exclusion criteria included corneal astigmatism greater than 2.0 diopters, pupil dilation of less than 5.0 mm, previous ocular surgery, previous ocular trauma, retinal and corneal pathology, chronic systemic corticosteroid or immunosuppressive therapy and immunocompromised patients, history of herpes zoster or herpes simplex ocular infection, and participation in any other ophthalmic drug or device trial. All the research and measurements followed the tenets of Helsinki and the study was approved by the local ethics committee, the Institutional Review Board of the Angeles University Foundation. Informed consent was obtained from all patients prior to the procedure, and all patients were recruited and operated at the Clinica Henson Eye, Ear, Nose, and Throat Center, Angeles City, Philippines.

Each patient received a study IOL (ActaLens™, Emmetrope, La Canada, CA) in one eye and a control IOL (CrystaLens® AO, B&L, USA, or an AcrySof IQ®, Alcon, USA) in the contralateral eye to allow for intraindividual comparison. The second eye was operated within 4 weeks' time. The first eye was randomized to the IOL type using an envelope method. Patients and examiners were masked to allocation, and the surgeon was masked to allocation until the time of IOL implantation.

**Figure 1:**
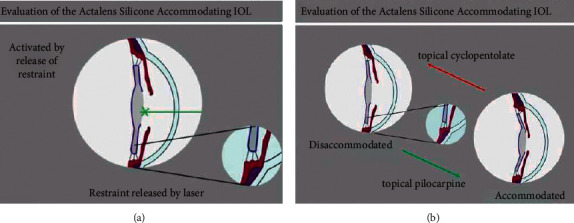
Drawing of the principle of the investigated IOL. Suture lysis (a) and effect of pilocarpine and cyclopentolate (b).

This lens is an axial displacement accommodative lens with a novel means of restoring zonular tension. Much like the CrystaLens, it exhibits a fully polymerized optic of fixed optical power. It is a single-piece silicone intraocular lens approximately eleven millimeters in length with a biconvex spherical optic 5.5 millimeters in diameter. The lens is similar in material and design to the USFDA-approved CrystaLens in that it is a silicone plate haptic lens with hinges between the silicone haptic and silicone optic portion. In addition, the ActaLens is composed entirely of MED 6820 silicone elastomer that was FDA approved for intraocular lenses in 1995. The main design innovation of this lens is that it is fabricated in a nonplanar accommodated configuration with two angulated haptics. During production, it is then flattened and held in a planar nonaccommodating configuration by a restraining suture made of Vicryl (Ethicon, USA). The lens is inserted in this prestressed, restrained configuration and allowed to attach to the capsular bag during the immediate postoperative period. Cycloplegia is maintained during the first four weeks of healing. The suture is released by laser suture lysis six weeks after surgery which allows the study IOL optic to move forward towards its accommodating nonplanar configuration, as shown in Figure 1. It is proposed that complete forward optic movement toward its as-manufactured shape is halted by the zonular system as zonular strain is reestablished. It is proposed that subsequent accommodation would lessen zonular tension and allow forward movement of the optic portion as the lens begins to flex. Conversely, disaccommodation and tightening of the zonules would pull the optic portion more posteriorly as the lens is once again pulled flat. With this design, it is suggested that forward excursion of the optic portion will be driven by the silicone hinge, rather than previous accommodative lens designs which rely on capsular elasticity for the driving force.

Prior to surgery, partial coherence interferometry technology (IOL-Master; Carl Zeiss Meditec AG, Germany) was used to measure the axial eye length of the eye and K-readings of the cornea. The SRK/T formula was used to calculate the IOL power, and the target refraction was emmetropia (0.0D to 0.5D).

All patients were operated upon using a standard small incision phacoemulsification technique by 2 experienced surgeons. The anterior chamber was filled with a viscoelastic device, and a continuous curvilinear capsulorrhexis was created of a size to allow a 360° rhexis-IOL overlap after IOL implantation. After hydrodissection and phacoemulsification, the surgeon was unmasked to the IOL type. The folded IOLs were implanted in the bag with the dedicated injector devices. After IOL implantation, care was taken to completely remove the viscoelastic device from behind the IOL, the bag, and the anterior chamber. Postoperative treatment consisted of PredForte 1% (prednisolone acetate) and Zymar (gatifloxacin 0.3%) eye drops 4 times daily for 4 weeks. Atropine 1% was given twice daily for the first four weeks after surgery.

For this study, an independent external observer (NH) performed all measurements at the 20-month follow-up. Therefore, two measurement days (with 48 hours in between) were set to measure all eyes before and 30 minutes after instilling 2% pilocarpine (twice, 5 minutes apart) on the first day and 1% cyclopentolate (twice, 5 minutes apart) on the second measurement day, respectively.

All eyes were measured with a Lenstar (Haag-Streit AG, Switzerland) and according to the group refractive index, the type of IOL (either silicone or acrylic) was used. In those cases, where clinically relevant posterior capsule opacification was observed, an Nd : YAG laser capsulotomy was performed after the study-related measurements, and the effect of the capsulotomy was assessed by remeasuring the patients 30 minutes after treatment again.

In addition, PCO was graded by two examiners independently at the slit lamp. Therefore, a grading system from 0 (no PCO) to 3 (Nd : YAG laser capsulotomy needed) was used. PCO analysis was then performed using the mean between both observers.

### 2.1. Statistical Analysis

For statistical analysis, Microsoft Excel 14.2.0 for Mac (Microsoft, USA) with a Statplus:mac version 5.8.3.8 plug-in (AnalystSoft, USA) was used, as well as SPSS 21.0 for Mac (IBM, USA). Descriptive data are always shown as mean, standard deviation (SD), median, and range. For bilateral comparison, the paired *t*-test and the Wilcoxon signed-rank test were used (depending on the fact if the results were normally distributed or not). Nonmetric data were compared using a chi-square test. Furthermore, scatter plots, box plots, and error bars were used to compare the two different IOLs.

For statistical modelling, partial least squares regression (PLS) was performed with Xlstat 2012. The advantages of PLS regression are explained elsewhere; here, we only want to introduce the outcome variables of PLS regression. Variable importance for projection (VIP) measures the importance of an explanatory variable to explain the dependent variable (the suggested thresholds of the VIPs : VIP values of 1.0 or more mean that it highly influences the regression model). To evaluate the regression model, a bootstrap method was used to estimate the weighting of each explanatory variable. The result of this bootstrapping method is shown in standardised coefficients (=beta coefficients) plots. For interpretation purposes, the larger the absolute value of a coefficient, the larger the weight of the variable, and if the confidence interval (whiskers) includes 0, the weighting of the variable is not significant.

## 3. Results

In total, 16 eyes of 8 patients were included. At the 20-month follow-up, the mean age of all patients was 69.0 years (SD: 4.0; range: 61 to 74 years). The female-to-male distribution was 3 : 5. Optical biometry data for each eye are depicted in Table 1. In none of the cases, a significant difference was observed between the groups (AL and CCT *p* > 0.99 and for Kmean *p*=0.294, respectively). All patients received a study IOL in one eye, and 4 patients received a CrystaLens in the control eye and 4 patients received an AcrySof IOL. In none of the cases, an intraoperative or postoperative surgery-related adverse event occurred.

### 3.1. Pilocarpine-Induced Effect

ACD measurements were possible in all eyes with an ActaLens and all eyes with an AcrySof IOL, but no or only insufficient peaks were measured in the case of the CrystaLens IOL. Therefore, all CrystaLens IOL eyes had to be excluded from the analysis.

**Figure 2:**
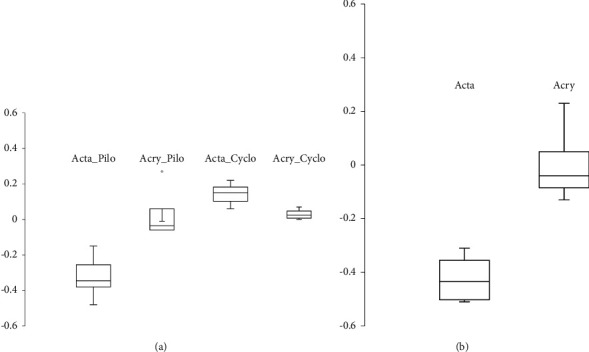
(a) Pilocarpine and cyclopentolate shift for the two investigated IOLs. (b) Calculated difference between pilocarpine and cyclopentolate shift for both investigated IOLs.

In the study group, the baseline and post-Pilocarpine ACD were 3.51 mm (SD: 0.68; median: 3.26; and range: 2.8 to 4.86) and 3.19 mm (SD: 0.77; median: 2.86; range: 2.39 to 4.71), respectively. This difference was found to be statistically significant (*p*=0.014, Wilcoxon signed-rank test) (Figures 2(a) and 2(b)). In all cases, the IOL shifted forward, and the mean ACD shift was 0.32 mm (SD: 0.12; median: 0.45; range: 0.15 to 0.48).

In the control group, the baseline and post-Pilocarpine ACD were 4.18 mm (SD: 0.40; median: 4.10; range: 3.79 to 4.74) and 4.22 mm (SD: 0.37; median: 4.20; range: 3.73 to 4.73), respectively. This difference was not found to be statistically significant (*p*=0.854, Wilcoxon signed-rank test) (Figure 1). The mean pilocarpine-induced ACD shift was 0.04 mm (SD: 0.16; median: 0.04; range: −0.06 to +0.27). In one case, a backward shift of 0.27 mm was measured.

### 3.2. Cyclopentolate-Induced Effect

In the study group, the baseline and postcyclopentolate ACD were 3.50 mm (SD: 0.68; median: 3.24; range: 2.84 to 4.84) and 3.65 mm (SD: 0.70; median: 3.37; and range: 2.90 to 5.00), respectively. This difference was found to be statistically significant (*p*=0.014, Wilcoxon signed-rank test) (Figure 1).

In all cases, the IOL shifted backward, and the mean ACD shift was 0.14 (SD: 0.06; median: 0.15; range: 0.06 to 0.22).

In the control group, the baseline and postcyclopentolate ACD were 4.02 mm (SD: 0.25; median: 3.95; range: 3.79 to 4.41) and 4.05 mm (SD: 0.28; median: 3.97; range: 3.79 to 4.48), respectively. This difference was not found to be statistically significant (*p*=0.181, Wilcoxon signed-rank test) (Figure 2). The mean cyclopentolate-induced ACD shift was 0.03 mm (SD: 0.03; median: 0.03; range: 0 to 0.07).

### 3.3. Predicting the Induced ACD Shift

**Figure 3:**
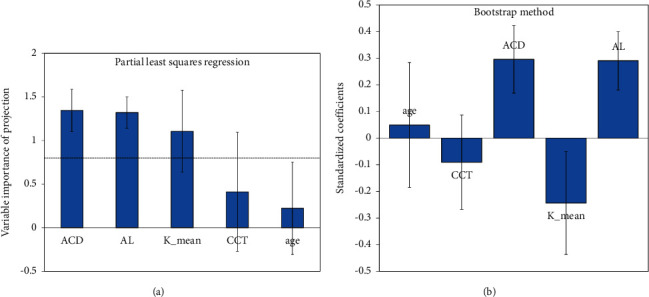
Partial least squares regression model and bootstrap model to predict the pilocarpine-induced ACD shift using AL (axial eye length in mm), Kmean (mean keratometry in diopters), CCT (central corneal thickness in *µ*m), and ACD (baseline anterior chamber depth in mm).

In the partial least squares regression model, the baseline ACD, the AL, and the Kmean values were found to be good predictors for the pilocarpine-induced ACD shift, whereas CCT and age were not found to be good parameters (Figures 3(a) and 3(b)).

(1) $$\begin{array}{c} {\text{ACD}}_{\text{shift}}=-0.65+0.05{\text{ACD}}_{\text{baseline}}-0.02{\text{K}}_{\text{mean}}+0.04\text{AL,} \end{array}$$

see also Figure 4.

Concerning the cyclopentolate-induced ACD shift, none of the investigated factors showed to have a significant predictive power concerning the ACD shift (only AL was found to have a VIP of 1.5, but the interpatient deviation in the bootstrapping model was too large to be considered a valuable predictive parameter).

### 3.4. PCO and Nd : YAG Laser Capsulotomy-Induced Effect

Least PCO was observed in the AcrySof IOL group (2 × 0.5 and 2 × 1), followed by the CrystaLens IOL group (3 × 2 and 1 × 1), and most PCO in the ActaLens IOL group (1 × 1.5, 2 × 2, 3 × 2.5 and 2 × 3), respectively. The bilateral comparison did not show a significant difference (AcrySof vs. ActaLens *p*=0.100 and CrystaLens vs. ActaLens *p*=0.174), respectively. In two of the ActaLens IOL group eyes, dense PCO was observed, and in both cases, a Nd : YAG capsulotomy was performed after assessing the cyclopentolate-induced ACD shift. Thirty minutes after the laser treatment, the optical biometry measurement was repeated. The ACD shift was +0.05 mm and +0.1 mm, respectively.

## 4. Discussion

The aim of this study was to investigate a novel accommodating IOL concept, attempting, for the first time, to restore zonular tension through a mechanism independent of capsular elasticity. Previous accommodative lens designs have posited either vitreous pressure or persistent capsular forces as a mechanism of restoring zonular tension and accommodative effect. After two decades of limited success relying on capsular remnants, this study lens introduces a method which may allow movement to be driven by a more robust elastomeric hinge. The ActaLens IOL consists of a single-piece, nonplanar silicone IOL that is prepared using a Vicryl suture to flatten and elongate the IOL. Flattening the study IOL causes stress to build in the hinge region, which is proposed to be the driving force to restore zonular tension and permit forward movement of the optic. After the IOL is stable in the capsular bag some weeks after surgery, the restraint is released. At the moment the restraint is removed by a laser, it is believed that the optic is urged forward by the relaxing hinges until this anterior force is equalized by counterbalancing elastic forces found in the posterior insertion of the ciliary muscle and the posterior zonular fibers. One proposed benefit of the mechanism of the study lens is that it may be more forgiving of varying sizes of the capsular bag following cataract removal as well as lost zonular tension during the capsulotomy. This may interfere with the ability of fluid-based and optic-shift-based lens designs to consistently reestablish zonular tension. Variable and increased capsular diameters immediately prior to IOL insertion may cause relative zonular laxity as the IOL is inserted. In addition, ongoing capsular changes such as capsular phimosis and lens epithelial migration may make stable capsular elasticity an elusive requirement for any accommodative lens design. With the proposed mechanism of the study lens, zonular tension would be created as a function of the design of the hinge and may be less capsule-dependent.

With accommodation and ciliary body constriction in the proposed mechanism, zonular tension should be reduced and the optic should be able to move forward. Disaccommodation would occur as the ciliary body relaxes and moves more posteriorly, retightening the zonules and pulling the study lens back into a more planar configuration.

A pilocarpine-induced shift was seen in all eight study eyes, with an average induced shift of 1/3 of a millimeter, or 1 diopter in an emmetropic eye, and about ½ of a millimeter between cycloplegic and pilocarpine-induced positions. These findings were significant, although the sample size was relatively low. For the CrystaLens, measurements with the Lenstar were not possible, as in none of the cases both peaks (anterior and posterior surface of the IOL) could be detected. In two cases, one peak was detected which did not change after pilocarpine instillation, but due to the uncertainty of the peaks, data were excluded from the analysis. In the AcrySof IOL group, no movement was observed except in one patient, where the ACD became deeper after pilocarpine instillation. Due to the fact that the optical biometry measurements were good in this patient and it was confirmed that the same eye was measured at both time points, data were included in the analysis. The pilocarpine-induced ACD shift of the investigated IOL seems to be similar to studies from the early postoperative period for other single-optic IOL designs, such as the 1CU, which is made of foldable acrylic hydrophilic material and has four broad-based haptics, which are thinner towards the optic (“transmission element”). Findl et al. [5] conducted a randomized bilateral study with intraindividual comparison and found a mean forward shift of 314 *µ*m 3 months after surgery. Contrarily, Hancox et al. [6] observed little forward shifting of the 1CU with accommodation using partial coherence interferometry (PCI) in contrast to a backward movement of a standard control IOL. In addition, the accommodative effect of the 1CU was found to be reduced at later follow-ups, beginning 1 year after surgery [7]. Two other investigations with observation periods of two years [8] and three years [9] had similar findings. This is contrary to the current study IOL, where the pilocarpine-induced ACD shift was still present 20 months after surgery. The decrease in accommodation over time was also observed in other single-piece accommodating IOLs, such as the OPAL-A (Human Optics AG, Germany) one-piece acrylic focus shift accommodating IOL with 4 flexible closed-loop haptics. Cleary et al. [10] showed a moderate ACD shift 1 month after surgery of 306 *µ*m, but this effect decreased significantly at the 6-month follow-up. Other accommodating IOLs showed lower pilocarpine-induced ACD shifts, such as the BioComFold (Morcher GmbH, Stuttgart, Germany), a single-piece hydrophilic acrylic with an outer, discontinuous ring and broad, perforated, and angulated haptics. Findl et al. [11] found a mean anterior shifting of only 0.116 mm for the model 43A and 0.222 mm for the model 43E evaluated with the PCI technique. Another accommodating IOL with no relevant pilocarpine-induced ACD shift is the CrystaLens IOL (C&C Vision later Eyeonics, now Bausch& Lomb Inc., Rochester, New York, USA). In a randomized controlled trial, it was shown that the CrystaLens IOL was not performing better compared to monofocal IOLs. In the same study, it was also shown that the optic of the CrystaLens IOL was moving in the wrong direction (backward instead of forward) [12]. A comparison with accommodating IOLs using other principles is difficult. One example is the Tetraflex IOL (KH-3500; Lenstec, St Petersburg, Florida, USA), which was shown to have a very low to moderate ACD shift [13, 14], but due to the additional alteration of the optical shape during accommodation, higher order aberrations are increased, resulting in a higher depth of focus [15]. Other accommodating IOLs, such as the NuLens (Herzliya Pituach, Israel), FluidVision IOL (PowerVision Inc., Belmont, CA, USA), the Synchrony IOL (Visiogen, Abbott Medical Optics, Santa Ana, CA, USA), the Lumina IOL (AkkoLens), the Sarfarazi (EA IOL, Bausch and Lomb, Rochester, NY, USA), or the Sapphire IOL (Elenza), use different concepts and a direct comparison is difficult.

Advanced regression modelling and bootstrapping showed that age was not one of the limiting factors (as well as central corneal thickness), but axial eye length, mean keratometry readings, and baseline anterior chamber depth were good parameters to predict the pilocarpine-induced ACD shift. Although the regression model showed strong predictive power, it has to be taken into account that the age range in this study was relatively small, and so was the sample size. However, PLS regression was used for small sample sizes, and the predictive power was good.

## 5. PCO

The investigated IOL showed a high PCO rate at the 20-month follow-up, and in two cases, an Nd : YAG laser capsulotomy was performed immediately after the last study-related measurement was performed. As previous studies showed, silicone IOLs do not tend to have more PCO compared to acrylic IOLs, so it is most likely that the increased PCO rates are the result of a nonsharp optic edge. [16].

Like earlier accommodating lens designs, this zonular tension restoring IOL showed moderate and consistent pilocarpine-induced forward shift of the optic. Unlike earlier accommodating lens designs, however, the study IOL demonstrates a forward shift that persists 20 months after implantation. What role the capsular independent mechanism plays in this persistence of effect is not known and will require further study. In addition, the investigated IOL seems to have a higher PCO rate compared to standard monofocal IOLs, but the effect of the Nd : YAG laser capsulotomy was minor and most likely did not decrease the accommodative power of the IOL.

## Figures and Tables

**Figure 4:**
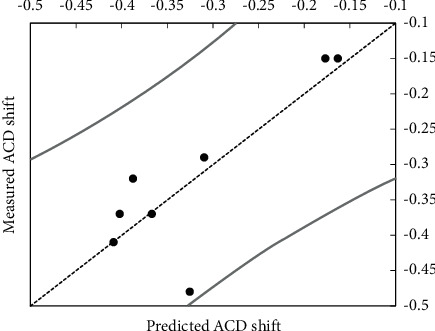
Correlation between the predicted pilocarpine-induced ACD shift using formula (1) and the really measured ACD shift in mm. The curved lines represent the 95% confidence interval.

**Table 1:** Optical biometry data.

	AL_od	AL_os	CCT_od	CCT_os	Kmean_od	Kmean_os
Minimum	21.94	21.90	498	489	41.47	41.41
Maximum	24.32	24.29	544	537	46.46	46.79
Median	23.00	22.88	505.5	504.5	44.99	45.12
Mean (SD)	23.04 (0.85)	23.01 (0.87)	510.5 (14.7)	505.4 (14.4)	44.42 (1.68)	44.56 (1.78)

AL = axial eye length in mm; CCT = central corneal thickness in *µ*m; Kmean = mean keratometry data in diopters; od = right eye; os = left eye.

## Data Availability

All data are presented in the manuscript and the figures.
